# Becoming and being a biobank donor: The role of relationships and ethics

**DOI:** 10.1371/journal.pone.0242828

**Published:** 2020-11-23

**Authors:** Signe Mezinska, Jekaterina Kaleja, Ilze Mileiko

**Affiliations:** Institute of Clinical and Preventive Medicine, University of Latvia, Riga, Latvia; Imperial College Healthcare NHS Trust, UNITED KINGDOM

## Abstract

Relational aspects, such as involvement of donor’s relatives or friends in the decision-making on participation in a research biobank, providing relatives’ health data to researchers, or sharing research findings with relatives should be considered when reflecting on ethical aspects of research biobanks. The aim of this paper is to explore what the role of donor’s relatives and friends is in the process of becoming and being a biobank donor and which ethical issues arise in this context. We performed qualitative analysis of 40 qualitative semi-structured interviews with biobank donors and researchers. The results show that relatedness to relatives or other types of close relationships played a significant role in the donors’ motivation to be involved in a biobank, risk-benefit assessment, and decisions on sharing information on research and its results. Interviewees mentioned ethical issues in the context of sharing relatives’ health-related data for research purposes and returning research findings that may affect their relatives. We conclude that the question of what information on family members may be shared with a biobank by research participants without informed consent of those relatives, and when family members become research subjects, lacks a clear answer and detailed guidelines, especially in the context of the introduction of the European Union’s (EU) General Data Protection Regulation. Researchers in Latvia and EU face ethical questions and dilemmas about returning research results and incidental findings to donors’ relatives, and donors need more information on sharing research results with relatives in the informed consent process.

## Introduction

Ethics codes regulating biobanking usually call for the promotion of public interest by emphasizing that research biobanks “should contribute to the benefit of society, in particular public health objectives” [1]. No doubt that social benefit is one of the cornerstones of the idea of research biobanks; however, as shown by an qualitative research review, “donation to biobanks is a complex process shaped by donors’ embeddedness in a number of social contexts” [2]. One example of neglecting this complicity is downplaying the role of relatedness, relationships, as well as reciprocity, which are important for many donors in the decision-making on biological sample donation to a research biobank and assessment of the risk-benefit ratio [3]. Potential biobank donors may not always be ready to reflect on the aims and benefits of biobanking in broad generalizations, such as public benefit, scientific knowledge, and communities of future patients. For some potential donors it is easier and more meaningful to evaluate the risk/benefit ratio of participation in a biobank within the context of personal benefits, or benefits for their relatives or friends who in some cases might be even research staff members.

Concepts of ‘relatedness’ and ‘relation’ are closely connected to reflection on relational elements of autonomy, which becomes especially important regarding genetic information where it is very complicated to separate a person from her relatives. Relational aspects can also not be ignored when thinking about health data in general and their use in biomedical research. In recent decades, many authors (most visibly feminist scholars and communitarians) have criticized the “hyper-individualism of the conception of autonomy” [4] which is used broadly as one of the central principles in reflection on ethical aspects of medicine and medical research. Instead, several authors suggest using the concept of ‘relational autonomy’ which is based on the concept of a person as “a free, self-governing agent who is also socially constituted and who possibly defines her basic value commitments in terms of interpersonal relations and mutual dependencies” [4].

Recognizing the importance of relational autonomy, including relatives or friends in the decision-making regarding participation in a biobank and involving family members’ interest in sharing research results with them, leads to specific ethical problems. There are some international ethics guidelines including at least minor references to the relational nature of autonomy, e.g. the Council for International Organizations of Medical Sciences (CIOMS) guidelines mention that potential research participants must be given adequate time for decision-making, including “time for consultation with family members or others”. [5] The CIOMS guidelines also remind of the moral duty to respect privacy, “including the precautions that are in place to prevent disclosure of the results of a subject’s genetic tests to immediate family relatives without the consent of the subject” [5]. At the same time, other influential ethics documents in the field of biobanking, such as the World Medical Association (WMA) Declaration of Taipei, do not mention relational aspects of autonomy at all, describing biological samples and related information in purely individual terms: “a sample obtained from an individual human being, living or deceased, which can provide biological information, including genetic information, about that individual” [1].

The aim of this paper is to explore what the role of donor’s relatives and friends is in the process of becoming and being of a biobank donor and what ethical issues arise in this context.

## Materials and methods

To describe the research process and results we applied the Consolidated Criteria for Reporting Qualitative Research (COREQ) [6]. The study is based on the analysis of 40 qualitative semi-structured interviews:

1. 20 interviews with 21 adult donors of the two largest research biobanks in Latvia: the Genome Database of Latvian Population and the biobank of the Institute of Clinical and Preventive Medicine, University of Latvia. The inclusion criteria were as following: (i) the person’s data and biological samples were stored in one of the biobanks and (ii) informed consent included the donor’s agreement to be re-contacted for further research purposes.
2. 20 interviews with researchers and clinicians. The inclusion criteria were as follows: (i) the person, as a professional, has been involved in the process of collection of biological samples for a biobank, or (ii) the person has worked as a researcher in a biobank-based research study.

Purposive sampling was used to cover the diversity of biobank donors. A biobank staff member contacted donors who had previously agreed to be re-contacted for further research studies and then invited them to participate in our study. In total, twenty-one donors participated in the interviews. One of the interviews involved two donors who were a married couple. The donor interviewees represented both genders, different age groups, different educational backgrounds, donors with different health status, and all geographical regions of Latvia. Eight interviewees were men and thirteen were women; their ages varied from 23 to 68 years. More information on participants and interviews can be seen in Table 1.

**Table 1:** Characteristics of donor interviewees and interviews.

Gender	Age	Education	Length of the interview	Interview code
Male	68	higher	0:44	M_68
Male	43	higher	0:31	M_43
Female	46	higher	0:31	F_46
Female	56	higher	0:37	F_56
Male	46	higher	0:39	M_46
Female	63	higher	0:36	F_63
Female	36	higher	0:37	F_36
Female	40	higher	0:36	F_40
Male	50	secondary	1:18	M_50_1
Male	51	secondary	0:29	M_51
Male	54	secondary	0:30	M_54
Male	50	secondary	0:54	M_50_2
Female	64	secondary, professional	0:21	F_64
Female	23	higher	0:35	F_23
Female	23	higher	0:33	F_23_2
Female	28	higher	0:24	F_28
female and male	43	higher	0:43	F_43_M_40
	40			
Female	49	secondary, professional	0:50	F_49
Female	58	higher	0:32	F_58
Female	62	higher	0:28	F_62

The donor interview guidelines were developed by the project team and consisted of six blocks: introduction, recruitment to the biobank, informed consent, privacy, usage of “old” collections of biological samples, and informing about research results. Interviews with donors were conducted between March and December 2019 by two experienced researchers. The length of donor interviews varied from 28 minutes to one hour and 18 minutes.

Purposive sampling was again used to choose researchers and clinicians for interviews. The interview guidelines for researchers and clinicians were developed by the project team and consisted of five blocks: introduction, collaboration with biobank(s), attitudes towards biobank(s), the needs of researchers and clinicians, and the process of knowledge creation. Interviews with researchers and clinicians were conducted between October 2019 and June 2020 by two experienced researchers. The length of the interviews varied from 32 minutes to one hour and 32 minutes. One half of interviews were conducted with senior researchers, seven with researchers and three with clinicians. More information on participants and interviews can be seen in Table 2.

**Table 2:** Characteristics of researcher and clinician interviewees and interviews.

Occupation	Length of the interview	Interview code
Senior researcher	1:32	SR_1
Senior researcher	0:38	SR_2
Researcher	0:37	R_3
Researcher	1:00	R_4
Senior researcher	0:50	SR_5
Researcher	0:50	R_6
Researcher	0:50	R_7
Senior researcher	1:02	SR_8
Senior researcher	0:37	SR_9
Researcher	1:10	R_10
Senior researcher	1:21	SR_11
Senior researcher	0:51	SR_12
Researcher	0:57	R_13
Clinician	0:36	C_14
Senior researcher	0:53	SR_15
Researcher	0:50	R_16
Clinician	0:32	C_17
Senior researcher	0:37	SR_18
Senior researcher	0:52	SR_19
Clinician	0:32	C_20

The research project was reviewed and approved by a Research Ethics Committee. All participants gave informed written consent to participate in the study. Interviews were audio recorded, transcribed verbatim and pseudonymized in the process of transcription in order to protect personal data. Interviews were analyzed using the qualitative data analysis program Nvivo 12. The coding was performed by all three authors. For data analysis, we applied thematic analysis in three stages, as suggested by Flick [7]. First, open coding was performed (the interviews were read line-by-line to reflect on the data and assist in the development of a codebook); secondly, all codes were reviewed and thematically similar codes were grouped in categories and were explored in terms of their relations to one another. In the third stage, all categories were reviewed to ensure that there was no overlap between them.

In the coding process of donor interviews we identified fifteen categories and seven of these were analyzed in the process of achieving the aim of this paper: (i) self-exploration, (ii) kinship, (iii) risk and benefit, (iv) research results, (v) motivation, (vi) informed consent, and (vii) relationships with research staff. Additionally, we analyzed one category from the coding of researcher and clinician interviews: kinship.

## Results

In interviews, the donors shared their experience of donating samples to a research biobank and participation in biobank-based research (some of the donors not only donated samples, but also participated in research studies requiring more involvement, e.g. completing additional questionnaires, changing a diet). The researchers and clinicians answered questions on collecting data on donor’s relatives and sharing research results with family members. The results showed that donors’ relatedness to relatives or other types of close relationships played a significant role in the motivation to be involved in a biobank, decision-making about participation, risk/benefit assessment and sharing information on research and its results. The donors mentioned three types of relationships influencing choices regarding participation in research biobanks and building the concept of relational autonomy: relationships with and relation to relatives, relationships with friends, and relationships with research staff.

### Decision-making and motivation

When donors considered participation in a biobank or biobank-related research study, they applied three different strategies regarding involvement of their family members in decision-making:

1. not to disclose the participation in a biobank to relatives because it was conceptualized as an individual choice and private information;
  Do you think that people who donate biological material containing genetic information should consult with their relatives or family?
  In general, I am a tight-lipped person, most likely I wouldn’t talk [about it]. Maybe if a conversation took a certain turn I’d let [them] know. I don’t know what I would do, maybe I would make an autonomous decision and not to share this decision. F_46
2. to inform relatives about their participation in a biobank or biobank-based study just to let them know about the decision;
  I didn’t discuss, I just told my mother that I have this [study], because it involves a diet and then you can’t eat what [your] mother gives you. Well, otherwise nothing, it was completely my decision and my family has never interfered in my business and my scientific activities. M_46
3. to discuss their potential participation in the biobank with relatives and make a decision together with them.
  Who helped [you] to decide?
  [My] family.
  So, you talked to [your] family?
  Of course. […] I have more or less responsibility also for them… as I am the children’s father. M_54

The choice to use one of these strategies can be linked to different reasons, circumstances and beliefs. In some cases, a decision whether to discuss participation in a biobank with relatives or not was linked to emotional closeness and quality of relationship. Sometimes, when the relatives were emotionally distanced or family members had different priorities or values (e.g. they did not trust the scientists), the donor decided not to disclose information about the donation of samples or participation in a study, even if the relationships were spatially close. In some cases, donors were not sure how relatives might respond or they were afraid that the response might be negative; therefore, they did not discuss the topic at all.

Did you discuss [donation] with your relatives?

No. Because they would say, “why is it needed?” and then start to talk me out [of it]. F_56

In situations where relationships were emotionally close, the interviewees more often let their relatives know about participation and discussed the decision to donate samples to a biobank or to participate in a study.

And was it important for you to discuss [donation] with relatives?

Yes, yes.

Why?

[…] it is a process involving me, and it is medicine—and nevertheless something is taken [from me] and removed. Anyway [they] should be informed. [My] wife had no objections, she is my partner, we are not officially married, but she had no objections. M_50

Another aspect impacting decisions to share and discuss information on donation to a biobank with relatives was spatial distance which may make communication between relatives less intensive. One of the interviewees explained that when she meets relatives they do not discuss all aspects of life, but only the most important, and that not may not include participation in a biobank and research studies.

How did your relatives perceive your choice to donate samples to the biobank?

[…] My relatives live in X [a regional city], I live in Riga. They… I don’t know, maybe I’ve mentioned something, but they don’t know about it in detail, I suppose. F_28

Some donors believed that their biological samples stored in a biobank will be inherited by relatives after their death. They expressed worries that this fact might cause problems in the future because it might be complicated and frustrating for their relatives to make any decisions regarding their biological samples. While, according to the law, this inheritance assumption is wrong, nevertheless it was mentioned as one of the reasons not to inform relatives about donating samples to a biobank.

[…] if we inform them [relatives], it makes them responsible to decide on behalf of the deceased on an issue about which they are not even aware. It could be hard for them; they might start to worry. F_43_M_40

Another important part of decision-making is motivation. Our interviewees mentioned various types of motivation for donation to a biobank, including progress of science, development of new medicines, and a way to find out information about themselves. In several cases, a donor’s motivation for participation in a biobank was clearly not based on considerations about benefits for the broader community or society; instead donors emphasized their considerations about personal benefits and benefits to relatives or friends. Some donors said that if donation of a biological sample would not help to find a better treatment for them personally, participation in a biobank might help their children or other family members. In particular, when interviewees were participating in a biobank-related study on a specific disease affecting a family member or friend, it was mainly conceptualized not as serving science in general, but as support for this particular person. Further, in interviews with professionals these mentioned that potential benefit for family members is an important motivation for potential donors.

Well… they [donors] are more motivated by the fact that it is for the future, that it is an investment in the future. […] It certainly does not harm him, but it can help his children or grandchildren. C_20

In some cases, relationships with a person was a reason for agreeing to donate or participate in a research study because a refusal might be viewed as a lack of support to the relative or friend. For example, one interviewee who herself was a biobank donor explained that the decision by her relatives to donate samples and to participate in a biobank-based study on diabetes was linked to her illness, and none of the family members refused to participate because they viewed participation in the study as manifestation of care.

I didn’t even hear from my relatives that anyone would object. As far I know, everyone took part [in the study]. They would disagree maybe only if they couldn’t get to the place—due to job, just for some other reason. Not because he doesn’t want to give [the biological sample], he just cannot give it. F_23

When speaking about motivation to participate in a biobank, relatedness and relationships become visible also in other forms. In two cases, female interviewees admitted that participation in a biobank-related study together with their partners was an adventure. In one of these cases it was also viewed as a good opportunity to do tests for the husband’s medical condition and to motivate him to see a doctor. In another case a female interviewee admitted that she decided to participate in a biobank-based study to find out whether the arthritis both she and her mother have is an acquired or inherited disease:

I wanted to know. I can’t afford genetic tests. […] my mom and I have arthritis, I wanted to know how to confirm if it’s inherited or developed during my lifetime. F_56

In some other cases, relationships manifested in the process of decision-making about donation as a motivation to support a relative or friend who is a researcher or biobank staff member. Some interviewees indicated that they themselves or their relatives knew people working in a biobank, and donation of samples and personal data was meant as support for this related person.

I don’t know, X [a biobank employee] called me, I know her since she wrote her Master’s thesis. I know her, and then my son told me that it needs to be done—mum, you can do it. Well, then I agreed. F_63

In this case the mutual acquaintance and history of relationships were viewed as the basis for trust and motivation to participate in a biobank, and the invitation was based on a personal request. In donor interviews cases were also mentioned where the invited person not only participated in research and donated samples to the biobank, but also engaged in recruiting more donors by inviting his or her acquaintances, colleagues or family members.

### Sharing relatives’ health-related data for research purposes

When donating biological samples and personal data for a biobank, donors are sometimes asked about hereditary diseases, health status and for other as about the health of their relatives. Sharing information about relatives’ health with biobanks was discussed both in donor interviews and in interviews with researchers and clinicians. In donor interviews we asked questions about donors’ readiness to share this kind of information with a biobank to find out what donors’ arguments pro and contra such practices are and what ethical issues they might identify. Most of the donors reported their personal experience from interaction with researchers.

When they spoke with you, were questions about your family included?

Well, the questionnaire was quite large, including the family and everything, because there were about 200 questions, if I’m not mistaken, about health, relatives and other things. M_68

Most of the interviewees did not consider sharing of information about their relatives with a biobank to be an ethical problem, since genetic information is shared in its very nature, and information about health issues and illnesses is often discussed among relatives anyway. Only two of the interviewees said they would not share any information about living relatives, in order to respect their privacy:

Only [information] about myself and nothing more. When it comes to inheritance, then about ancestors, but I respect the personal space of my relatives. I am ready [to tell] about myself, but about others, [researchers] should talk with them. I wouldn’t like to involve them. M_68

Donors who stated that they would share information on their relatives often admitted that they would not ask for consent from them before sharing information on their health. Most did not see this as an ethical problem; however, some interviewees reflected on how the requirements on personal data protection and the principle of autonomy might be applicable in this context.

All I thought about was what the relatives or the distant relatives would say about the fact that I had made such information public. My sister…

Did you ask their permission?

I did not ask my sister, but I asked my mother a question about her illnesses and age [when the illnesses started]. We didn’t really discuss it. But I suppose that my relatives would not mind. F_43_M_40

In most of the interviews, donors expressed the view that relatives would not object to sharing their health information with a biobank and researchers. Some interviewees believed that they have a right to share such information if they do not mention a relative’s name as in this way they keep confidentiality and information may be considered anonymous.

Yes, I would provide [the information] as much as needed. For example, if it’s all confidential and I don’t have to reveal the names and surnames of my relatives […] then why not?

So, one may tell about relatives without identifying them?

Yes, exactly, why not? However, in fact, if you know my identity, you can probably find out all [the identities of relatives], but well… But well, if it is needed for science then I would probably provide as much information as is necessary. F_23

In the interviews, researchers and clinicians recognized the importance of information on the health of donors’ relatives but admitted that collecting personal information on donor’s relatives might be problematic because of personal data protection requirements.

What do you think, how much should be asked about relatives?

Well, [donors] should be asked at least about their first-degree relatives: have they had any kind of diseases that might be inherited? C_17

In some donor interviews, a breach of confidentiality was perceived by donors as a potential risk to their family.

I wouldn’t want it to appear in a newspaper–that family has the following hereditary illnesses. F_43_M_40

Donors and professionals viewed confidentiality and data security as essential tools for addressing various privacy risks, not only for donors themselves but also for their relatives; however, there was a lack of clarity in how to apply these tools when dealing with information about a relative’s health. Several researchers mentioned that, in their view, the information which is collected about donor’s relatives is not identifiable because the name of the relatives is not asked.

We will not identify relatives and who they are. SR_12

Some researchers said that there is sense in collecting information on relatives only in cases where genetic analysis is carried out. At the same time, other researchers expressed the view that the more information on donors’ relatives collected from a donor in any type of research the better. They hold the view that also lifestyle information on donors’ relatives and various socio-economic aspects may help to analyze, understand and interpret results of testing of biological samples. However, one of the possible problems mentioned by researchers and clinicians was the donor’s lack of knowledge about their relatives.

For example, in our routine questionnaires we ask whether their relatives, like their father’s mother, their mother’s mother, and so on, have had cardiovascular diseases, different types of cancer, other problems, since it is such essential information, and well, not everyone can answer such in-depth questions, for example, I couldn’t answer what significant problems my relatives have had, I assume I’m not the only one. R_10

Several researchers emphasized that in any case the information provided by donors on their relatives is not fully reliable and quality of data might be questionable.

### Sharing research results with relatives

We asked all interviewees several questions on the management of incidental findings and returning of research results, including those findings which might be meaningful and important for donors’ relatives. The interviewees had different ideas on how incidental findings and research results should be returned. Some donors believed that their moral obligation is to share the results and incidental findings with relatives. They emphasized that this knowledge might help prevent health problems for members of their family in the future, e.g. to avoid diabetes by changing lifestyle and diet and by starting necessary treatment.

You said that you discussed the results with your daughter, what did she change in her life?

Well, […] like me she also has a tendency for being overweight. My mother who was my height was 80 [kg], I am 65 kg and I’ve been told to lose weight. And I knew from my mom also that I have a tendency for diabetes. […]

Is there anything your daughter is going to change?

She is on a diet all the time, because she has [got] a tendency for being overweight not only from my side, but also from the other. F_62

Some donors were not sure how their relatives would perceive this type of information and whether their relatives would be willing to know it at all. In one case, the donor believed that it would be unethical for biobanks to share genetic information that would allow finding out from whom the person has inherited a disease because it could lead to the blaming of relatives. Some donors mentioned that the fact that a condition is treatable might be crucial in deciding about sharing research results or incidental findings.

Well, for a young child it is possible then to do gene sequencing to know if it [the disease] will be inherited […] Well, maybe about those [diseases] which can be treated there is a need to know, but about those which can’t be treated–no. F_56

Researchers and clinicians mainly shared the view that they do not have the right to inform donor’s relatives about incidental findings and research results.

In fact, the relative has not given us the right to contact him. In my view, we may only inform the person with whom we have initially met. Well, but that information… To some extent, it would be ethically right if it reached the relative. SR_12

Some researchers and clinicians recognized the importance of informing relatives about incidental findings and research results, but they also pointed to possible ethical challenges that could arise. At least two experts believed that genetic information poses a risk in uncovering “family secrets”, e.g. information about biological parents.

And then the researchers and scientists get into a dilemma how to tell it all, whether they have to tell it, whether they may say it and to what extent, and who will be responsible for disclosure of the information. Because then the other person can sue you.

Who exactly?

Well, for example, the woman–a mother who has been unfaithful to her husband. P_14

In this context, an unanswered question for professionals was also how to exercise relatives’ rights not to know about potential diseases or genetic risks that could affect them. Even in cases where a donor has given their informed consent to receive information on incidental findings, there is usually no consent given by the donor’s relatives. At the same time, some researchers and clinicians held the view that incidental findings that may seriously impact the health of donors’ relatives must be reported to them, especially if the risk is high.

If we clearly understand that this mutation is a very high-risk mutation leading to development of aortic aneurysm, and this is a hereditary mutation, the young man is at very high risk of tearing his aorta by the age of 30, and he knows nothing about it and likes extreme sports, I think it is a life-changing decision that he can take at that moment. […] These are very individual and very specific cases of course, but it can happen from time to time. R_16

Often researchers and clinicians did not have any idea of how it would be possible to inform donors’ relatives about incidental findings because they do not have their consent and contact information. This is why some said they would inform the donor and ask him/her to inform relatives.

[…] and then there is a question of whether a group of relatives should be informed?

I think it is necessary to inform the individual who is involved in the study, from whom the samples are taken. And it’s up to him whether to reveal to others. […] It’s his responsibility. He would also bear some criminal liability and other kinds of responsibility if, for example, a person dies, because of ignorance, because he has not been informed. P_14

It was also mentioned that putting responsibility for sharing information with relatives onto the donor may lead to practical problems, especially if their relationships with relatives are not good.

## Discussion

Biobanks are not unique in facing ethical issues arising from different forms of relationships; they are just another arena in which relatedness and relationships are becoming visible. Relationships with family members and ethical challenges were often referred to in the interviews when biobank donors spoke about decision-making, informed consent, research results, and confidentiality. Our research also shows that not only relatedness to relatives but also relationships with friends can build motivation to become a biobank donor as a manifestation of care, love and support while at the same time raising certain ethical questions.

One of the central ethical themes in this context is the sharing of information. As Strathern notes, “knowledge itself imposes an obligation on the knower in relation to those around him or her. It causes moral action and creates a compulsion to act” [8]. Sharing of information becomes an even more complicated task because involvement of relatives often shows the connection and disconnection already existing between family members which is directly linked to Strathern’s idea of regarding kinship as a system forming constant connection and disconnection [8]. The problem is how to adjust ethical guidelines to the reality of connection and disconnection between relatives.

The question what information on family members may be shared with a biobank by research participants without informed consent of those relatives, and when those family members become research subjects still lacks a clear answer and detailed guidelines, especially in the context of introduction of the General Data Protection Regulation in the EU. Our interviews showed that in current practice, researchers (and also donors) hold different views on what is ethically and legally acceptable within the EU.

The discussion on this topic in United States (US) and Canada has defined a difference between a ‘third party’, “an individual (or organization or institution) who is not a researcher or a subject, but who is affected by the relationship between those persons” [9], and ‘primary’ and ‘secondary research subject[s]’, where “[t]he research subject interacting with investigators and answering questions would be regarded as the primary research subject, and those relatives about whom identifiable private information is collected would be regarded as secondary research subjects” [9]. Another piece of terminology used with the same meaning is ‘active participant (proband)’ and ‘passive participant’ [10].

One of the attempts to develop recommendations regarding privacy of family members in research was suggested in the US by Botkin in 2001 as a reaction to the case at Virginia Commonwealth University where a father complained that information about him which his daughter has provided to researchers as a participant of a research study without his consent was sensitive and private [11]. This case started a wide-ranging debate in the US on privacy and rights of family members in pedigree and other types of research [10–14]. Botkin developed a list of questions that might be useful for research ethics committees and researchers in deciding when family members should be viewed as secondary research subjects in a research study and whether informed consent in certain cases can be waived by a research ethics committee [11]. Even further, some authors emphasized that research ethics committees “should carefully consider the impact of their research on subjects’ family members, even if those family members do not meet the technical definition of secondary subjects” [15]. A broad discussion in the EU on this topic is still lacking.

Some of the donor interviewees in our study emphasized not only possible benefits, but also risks to their relatives as third parties or secondary research participants. Donors’ reflection on risks to family members has been mentioned in many research studies, especially in the context of whole genome sequencing. For example, Robinson et al. mention a “primary care participant who withdrew due to insurance and privacy concerns [who] was particularly concerned about the impact of this information on her children’s future insurability” [16]. Possible risks are the main reason why some authors call for gaining informed consent from donors’ relatives for genome research by suggesting family-centric initiatives [16, 17]. At the same time, family-centric initiatives for informed consent raise several challenges, such as the definition of family, “conflicting wishes of family members”, risk of coercion, and the need for technical solutions and procedures [17].

Our study showed that researchers in Latvia face ethical questions and dilemmas about returning research results and incidental findings to donors’ relatives and this issue is not clearly regulated by law or guidelines in Latvia. In the scientific literature, there is a broad discussion on this topic leading to a consensus that researchers need more guidance and harmonization of requirements on return of research results to relatives of biobank donors [17–21]. Additionally, donors need more information on sharing research results with their relatives in the informed consent process. As in our study, other research studies also have shown that values of the family, as well as distance or closeness, may influence the views on sharing research results and should be taken into account when developing laws and guidelines. For example, Gordon et al. conclude that “policies governing return of genomic results should consider how families understand genomic data, how they value confidentiality within the family, and whether they endorse an ethics of sharing” [22] and Xuan Zhu et al. emphasize that “factors influencing family disclosure decisions included the family’s biological and emotional closeness” [21].

## Conclusions

For a portion of the donors in our study, considerations relating to benefits for relatives or friends is an important motivation behind the donation of samples and personal data to a research biobank. To become a donor or research participant in such cases is viewed as a manifestation of care or fulfilling a moral duty towards them.

Most of the interviewees did not consider the sharing of information about relatives with biobank to be a problem, as far as the names of the relatives are not mentioned. At the same time, questions of what information on family members may be collected from research participants without the informed consent of those relatives, as well as when family members themselves become secondary research subjects, still lack a clear answer, especially in the context of introduction of GDPR in the EU.

Researchers in Latvia lack proper guidance and need better harmonization of recommendations when facing ethical questions and dilemmas on returning research results and incidental findings to donors’ relatives. At the same time, donors need more information on sharing research results with their relatives in the informed consent process. It should be noted that this problem has not only local relevance, but actually applies to the whole of the EU.
